# The anti-tumor activity of pralatrexate (PDX) correlates with the expression of RFC and DHFR mRNA in preclinical models of multiple myeloma

**DOI:** 10.18632/oncotarget.27516

**Published:** 2020-05-05

**Authors:** Cristina Kinahan, Michael A. Mangone, Luigi Scotto, Michele Visentin, Enrica Marchi, Hearn Jay Cho, Owen A. O’Connor

**Affiliations:** ^1^ Columbia University Medical Center, Center for Lymphoid Malignancies, New York, NY, USA; ^2^ Department of Clinical Pharmacology and Toxicology, University Hospital Zurich, University of Zurich, Zurich, Switzerland; ^3^ Department of Hematology and Medical Oncology, Tisch Cancer Institute, Icahn School of Medicine at Mount Sinai, New York, NY, USA; ^*^ Co-first authors

**Keywords:** antifolate, multiple myeloma, pralatrexate, biomarker, reduced folate carrier (RFC)

## Abstract

Multiple myeloma (MM) is the second most common hematologic malignancy. While major advances have been made in the disease, it is still incurable. Although antifolate-based drugs are not commonly used to treat myeloma, new generation analogs with distinct patterns of preclinical and clinical activity may offer an opportunity to identify new classes of potentially active drugs. Pralatrexate (PDX), which was approved for the treatment of relapsed or refractory peripheral T-cell lymphoma in 2009, may be one such drug. Pralatrexate exhibits a potency and pattern of activity distinct from its predecessors like methotrexate (MTX). We sought to understand the activity and mechanisms of resistance of multiple myeloma to these drugs, which could also offer potential strategies for selective use of the drug. We demonstrate that PDX and MTX both induce a significant decrease in cell viability in the low nanomolar range, with PDX exhibiting a more potent effect. We identified a series of myeloma cell lines exhibiting markedly different patterns of sensitivity to the drugs, with some lines frankly resistant, and others exquisitely sensitive. These differences were largely attributed to the basal RFC (Reduced Folate Carrier) mRNA expression levels. RFC mRNA expression correlated directly with rates of drug uptake, with the most sensitive lines exhibiting the most significant intracellular accumulation of pralatrexate. This mechanism explains the widely varying patterns of sensitivity and resistance to pralatrexate in multiple myeloma cell lines. These findings could have implications for this class of drugs and their role in the treatment of multiple myeloma.

## INTRODUCTION

Multiple myeloma (MM) is a malignancy of immunoglobulin-secreting plasma cells, and is considered the second most common hematologic malignancy. Despite the introduction of many effective drugs over the past decades, the disease is widely considered incurable [1]. Incomplete eradication of the disease has been attributed, at least in part, to heterogeneity and clonal evolution of the malignant plasma cell population [2, 3]. Recent studies utilizing advances in single-cell sequencing and whole exome profiling have identified subclonal tumor cell populations present at initial treatment which expand over time, producing increasingly drug resistant phenotypes [4]. Identification of functional biomarkers which correlate with sensitivity or resistance to a particular drug or class of drugs is a principle component of the “precision medicine” approach to treat many malignant diseases, including relapsed multiple myeloma [5]. In theory, the integration of novel agents into a tailored treatment strategy based on the patients’ disease biology could increase the probability of favorable outcome.

Pralatrexate (PDX, 10-propargyl 10-deazaaminopterin) is a folate analogue rationally designed to have greater affinity (more than 10-fold greater affinity compared to methotrexate) for RFC, and has proven more potent than methotrexate (MTX) [6, 7]. The RFC transporter is an oncofetal protein shown to be more highly expressed on fetal and malignant tissue, and is the primary mechanism for internalization of the drug into the tumor cell. The activity of PDX in peripheral T-cell lymphoma (PTCL) likely goes beyond its effects as an inhibitor of dihydrofolate reductase (DHFR), a hypothesis supported by the observation that leucovorin can be given concomitantly with pralatrexate without compromise of its activity in both preclinical and clinical settings [8, 9]. Additionally, pralatrexate has greater affinity for folylpolyglutamate synthase (FPGS), which mediates polyglutamylation of the drug, leading to prolonged intracellular retention [10–13]. Pralatrexate inhibits tumor growth with more potency than other antifolates across a host of cancer cell models [14, 15]. In particular, pralatrexate has exhibited marked activity in T-cell malignancies in both the preclinical and clinical setting, which led to it becoming the first drug approved for patients with relapsed or refractory PTCL. The activity in PTCL appears out of proportion to what has been described in B-cell malignancies and solid tumors studied to date [16–24]. While the basis for this activity in PTCL is a matter of continued research, it raises the question as to why some malignant diseases exhibit such intrinsic resistance, while others an intrinsic vulnerability to the drug.

A number of pharmacologic determinants that correlate with methotrexate resistance have been established, including DHFR, FPGS, gamma-glutamyl hydrolase (GGH) and RFC [25–30]. The first demonstration of a relationship between one of these determinants and methotrexate resistance was established by Bertino and Shimke, who described gene amplification of DHFR as a mechanism of resistance to MTX in a colon carcinoma and acute leukemia cell lines [31–33]. Several papers have established a correlation between functional RFC protein expression and MTX sensitivity, including studies in human T-cell acute lymphoblastic leukemia cells, and solid tumor cell lines [34]. In one study, treatment with methotrexate reduced DHFR gene expression while increasing RFC mRNA in sensitive cell lines, which did not occur in MTX resistant cells [35]. While correlations with these pharmacologic determinants and drug sensitivity have been demonstrated in select disease settings for MTX, little to no data have established these determinants for pralatrexate in any biological setting.

Drug screens in our laboratory indicate that some myeloma cell lines exhibit marked sensitivity to PDX and MTX, while others maintain a more resistant phenotype. Based on these findings, we sought to better understand the mechanisms of intrinsic resistance and sensitivity, in anticipation of identifying strategies to optimize the drug in myeloma and other malignant diseases.

## RESULTS

### Comparison of MTX and pralatrexate cytotoxicity

**Figure 1:**
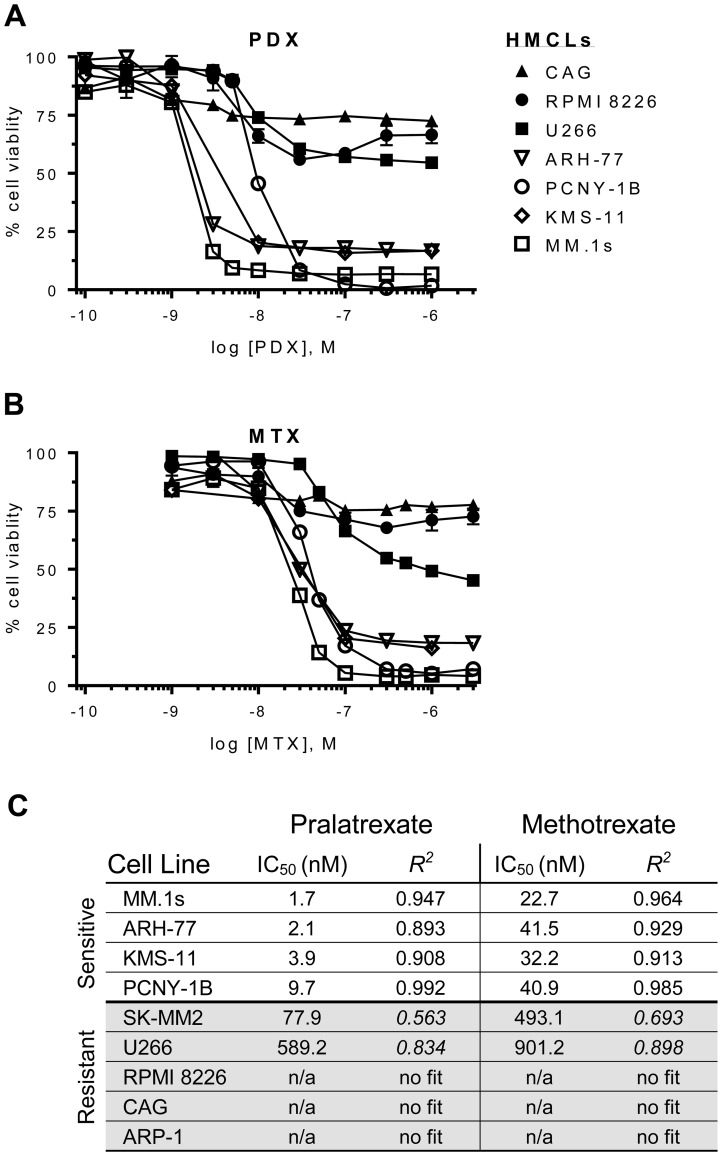
Dose-response curves for antifolates in a panel of HMCLs. Human multiple myeloma cell lines (HMCLs) were incubated with increasing concentrations of (**A**) pralatrexate (PDX) or (**B**) methotrexate (MTX) for 48 hrs. The HMCLs segregated into sensitive (*open icons*: ARH-77, MM.1s, KMS-11, PCNY-1B) or resistant (*filled icons*: U266, CAG, RPMI 8226) groupings. (**C**) An ordered list of half maximal inhibitory concentration (IC_50_) values for PDX and MTX in HMCLs. The values were determined using an ordinary least squares nonlinear curve fitting method and the goodness of fit was determined valid with an R^2^ > 0. The curves were normalized to untreated cells (100%) and bortezomib (10–50 nM) treated cells (0%). Data represent the mean ± SD of at least three experiments. Cell viability was determined by an ATP-dependent luciferase-based reporter assay.

Figure 1 presents the concentration effect relationships for MTX and pralatrexate in the panel of myeloma lines. Both MTX and PDX caused significant reduction in cellular ATP levels in a subset of myeloma cell lines, with pralatrexate appearing about a log more potent in sensitive lines compared to MTX. No cell line exhibited sensitivity to only one antifolate, with all lines either sensitive to MTX and pralatrexate, or resistant to both agents The myeloma cell lines appeared to segregate into two distinct groups based on their patterns of sensitivity. The myeloma cells lines MM.1s, ARH-77, KMS-11 and PCNY-1B exhibited marked sensitivity to both drugs, with pralatrexate exhibiting higher potency compared to MTX for all lines (PDX IC_50_: 1.7–9.7 nM vs MTX IC_50_: 22.7–40.9 nM; Figure 1A, 1B). Conversely, SK-MM2, U266, RPMI, ARP-1 and CAG cell lines exhibited marked resistance with very high IC_50_ concentrations or no sensitivity at all at the highest concentrations (Figure 1C). All responses in the sensitive myeloma cell lines were both time and concentration dependent (Supplementary Figure 1). While select lines exhibited cytotoxicity at 24 hours, the maximum effect was achieved at 48 hours, with no increase in cell death observed at 72 hours of incubation.

### Induction of apoptosis

**Figure 2:**
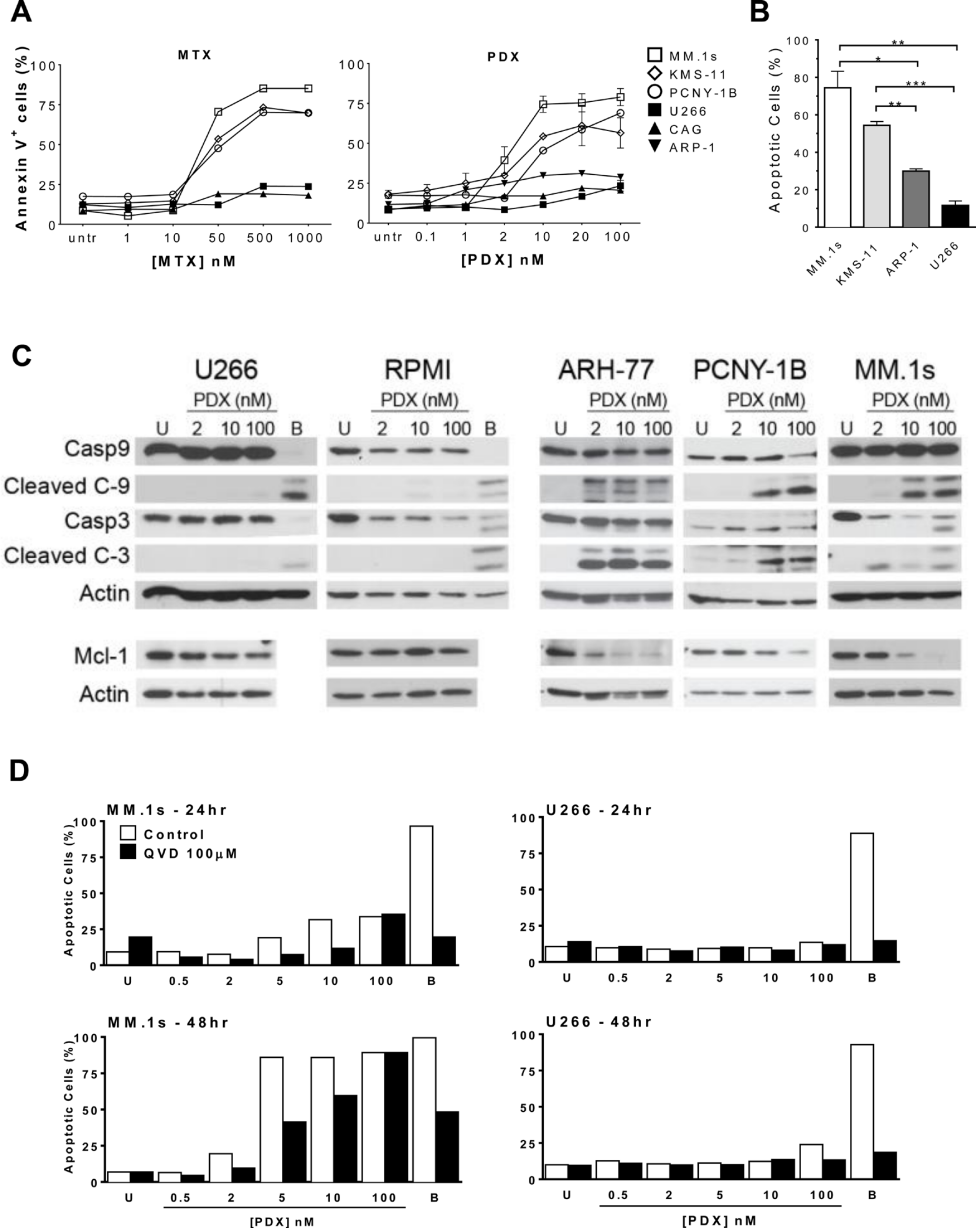
Antifolates induce apoptosis in a dose-dependent manner in sensitive HMCLs. (**A**) Sensitive HMCLs - MM.1s (*open square*), KMS-11 (*open diamond*), PCNY-1B (*open circle*) and resistant HMCLs – U266 (*filled square*), CAG (*filled triangle*), ARP-1 (*filled inverted triangle*) were incubated with increasing concentrations of either MTX or PDX for 48 hrs. Apoptosis was determined by flow cytometry using a fluorochrome-conjugated-AnnexinV marker. Untreated cells (*untr*) served as a negative control and bortezomib (10–50 nM) treated cells (*not shown*) served as a positive control for apoptotic cell death. (**B**) Sensitive HMCLs, MM.1s (*white*) and KMS-11 (*light gray*), exhibit more apoptosis than resistant HMCLs, ARP-1 (*dark gray*) and U266 (*black*), after 48 hrs of exposure to 10 nM PDX. The statistical analysis uses data from three separate experiments. Paired student *t*-test generated *p* values: ^*^ *p* < 0.02, ^**^ *p* < 0.005, ^***^ *p* < 0.001. (**C**) Western blot analysis depicting relative protein levels of full length and cleaved caspase 9, caspase 3, full length Mcl-1, and β-actin. HMCLs (U266, RPMI 8226, ARH-77, PCNY-1B, and MM.1s) were treated with 2, 10 or 100 nM PDX for 48 hrs. Artifact observed in U266 10 nM PDX treated Actin sample. When the X-ray film is pulled out it can cause scratches to appear on the film, causing such artifacts. (**D**) MM.1s (*left* panels) and U266 (*right*) were incubated with increasing amounts of PDX (0.5 nM – 100 nM) in the presence (*black* bars) or absence (*white*) of 100 μM of pancaspase inhibitor Q-VD-OPh (QVD). Data were collected at 24hrs (*top* panels) and 48hrs (*bottom*) of incubation. U = vehicle control cells, B = bortezomib treated cells (10 nM).

Loss of cell viability was corroborated by examining induction of apoptosis in drug-treated cells through Annexin V and caspase staining. In general, the same patterns of sensitivity and resistance noted in the cell viability experiments were observed in the apoptosis assays. Across all cell lines studied, pralatrexate was more potent than MTX, and no cell line exhibited sensitivity to only one of the two drugs studied. For example, as shown in Figure 2A, MM.1s cells exhibited a concentration dependent induction of apoptosis to both MTX and pralatrexate, with the latter occurring at about a log lower than what was observed for MTX. Treatment of MM.1s, KMS-11 and PCNY-1B cells with increasing concentrations of pralatrexate or MTX for 48 hours resulted in a concentration dependent increase in apoptosis, quantified as the AnnexinV^+^ cell population (Supplementary Figure 2A, 2B). Again, the same cell lines classified as sensitive in the Cell Titer Glo Assay were sensitive in the apoptosis assays. The resistant myeloma cell lines, including U266, CAG and ARP-1 exhibited no significant increases in apoptosis compared to vehicle treated controls across a broad concentration range of either MTX or pralatrexate. The differences observed in apoptotic cell numbers between sensitive cell lines (MM.1s and KMS-11) and resistant cell lines (U266, ARP-1) were significant at 48 hours after exposure to 10 nM PDX (Figure 2B). These data corroborate the cytotoxicity results confirming the increased potency of pralatrexate compared to MTX in antifolate-sensitive myeloma cell lines.

We investigated the mechanisms of apoptosis by examining the activation of pro-apoptotic proteins in pralatrexate treated myeloma cell lines. After 48 hours of pralatrexate exposure, the sensitive cell lines MM.1s, ARH-77 and PCNY-1B exhibited a concentration dependent cleavage of caspase 3 and caspase 9, key components of the cell-intrinsic apoptosis pathway, compared to untreated negative controls (Figure 2C, Supplementary Figure 2C). Exposure to 2 nM, 10 nM or 100 nM of pralatrexate did not induce any cleavage in the resistant cell lines RPMI and U266. In addition, treatment with pralatrexate caused a decrease in the anti-apoptotic long form of Mcl-1 protein in sensitive cell lines. The Mcl-1 protein, a Bcl-2 family member, has been demonstrated to be particularly important for the survival of myeloma cells [36, 37]. In the presence of increased pralatrexate concentrations, the expression of the 40 kDa isoform of Mcl-1 decreased in MM.1s, PCNY-1B and ARH-77 cells (Figure 2C). While the responses were concentration dependent, the range of Mcl-1 reduction spanned from total elimination (MM.1s) to moderately lower levels as seen for PCNY-1B. Incubation with pralatrexate did not alter the relative quantity of Mcl-1 in RPMI and U266 cell lines, which is consistent across the cytotoxicity and apoptosis data. The addition of the pan-caspase inhibitor QVD (QVD-OPh: quinolyl-valyl-O-methylaspartyl-[-2, 6-difluorophenoxy]-methyl ketone) diminished the level of apoptosis induced by PDX in the sensitive cell line MM.1s (Figure 2D), producing no effect on apoptosis in the non-responsive, resistant cell line U266.

### Pralatrexate treatment blocks S-phase cell cycle progression in sensitive myeloma cell lines

**Figure 3:**
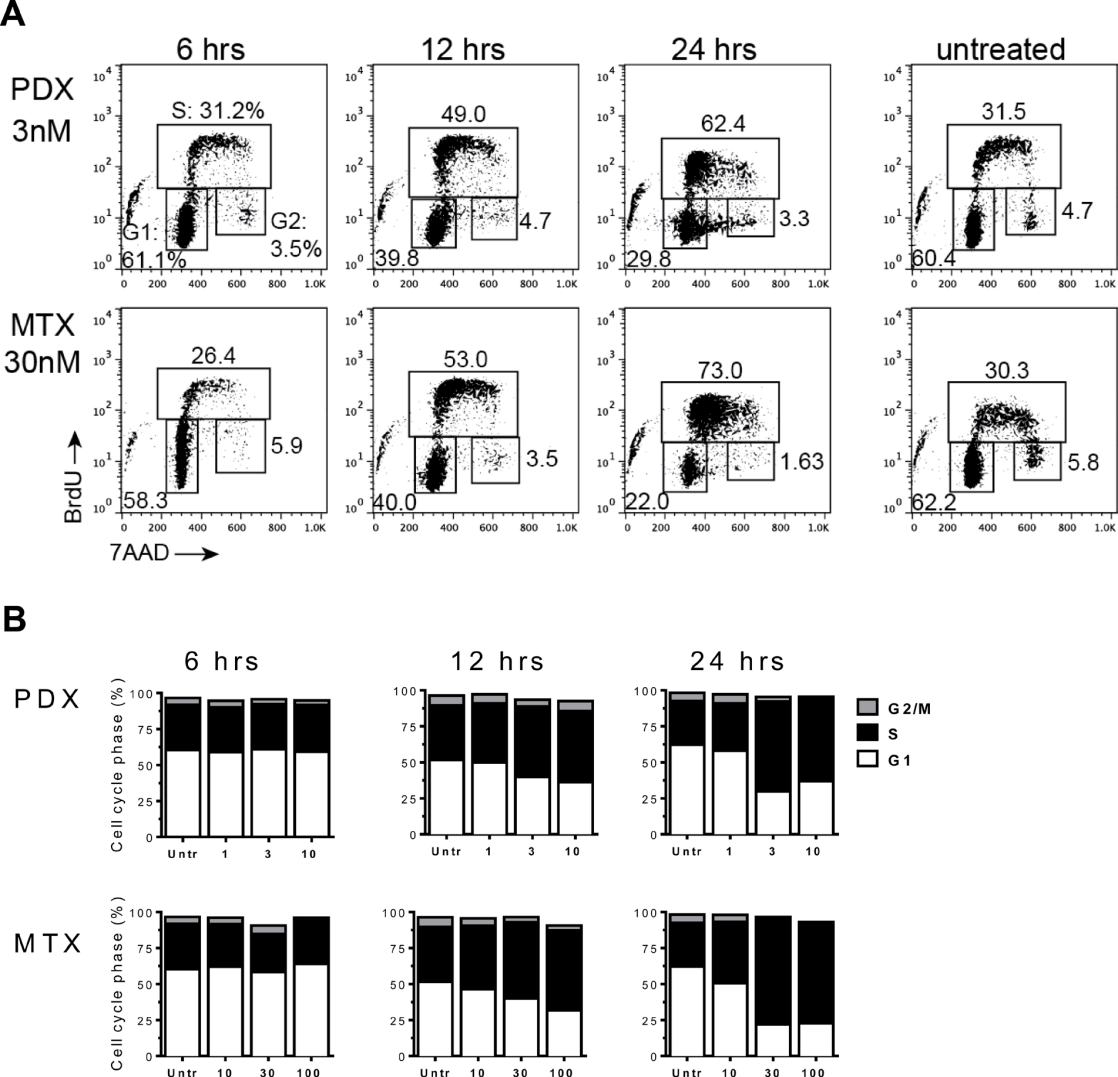
Antifolates cause cell-cycle disruption in MM.1s myeloma tumor cells. MM.1s cells were incubated with either PDX (1 nM, 3 nM, 10 nM) or MTX (10 nM, 30 nM 100 nM) for 24 hrs. Time points were taken at 6, 12 and 24 hrs. Thirty minutes prior to isolation at each time point MM.1s cells were pulsed with Bromodeoxyuridine (BrdU). (**A**) A flow cytometry dot plot array shows 7AAD/αBrdU co-stained MM.1s cells at 6 hrs, 12 hrs, and 24 hrs at single dose - PDX (3 nM) and MTX (30 nM). The gates define cells in the three stages of the cell cycle: G1, S-phase (S), and G2/mitosis (G2). (**B**) A graphical representation of the entire experimental data set. Drug concentrations listed on the x-axis are in nM.

As shown in Figure 3A, MM.1s cells treated with MTX or PDX exhibited a distinct pattern of cell cycle events compared to untreated cells as early as 12 hours after exposure to the drug. Pralatrexate or MTX treated MM.1s cells accumulated in early G1/S phase transition, as demonstrated through 7-AAD and Bromodeoxyuridine (BrdU) co-staining (Figure 3A). Drug-treated MM.1s cells were able to initiate DNA synthesis, visualized as an increase in incorporation of pulsed BrdU (S-phase). However, the cells were unable to progress through S-phase, as visualized as by an increase in BrdU^+^ diploid (2n) cells. This effect was time and concentration dependent (Figure 3B). The cell cycle analysis of resistant U266 cells was unaltered following treatment with PDX or MTX (1, 3, 10 nM – data not shown). Interestingly, a similar effect was reported by Ramirez *et al*. who demonstrate resistance of U266 cells to the multi-targeted antifolated pemetrexed [38]. Across all sensitive cell lines, pralatrexate induced cell cycle arrest in a concentration dependent manner. These findings confirm the cell viability and apoptosis data above, demonstrating that the patterns of sensitivity and resistance remain intact across the assays, and that pralatrexate is superior to MTX in all assays.

### The microenvironment does not alter patterns of sensitivity or resistance

Under normal host conditions, the bone marrow microenvironment contributes to the maintenance and progression of multiple myeloma tumor cells through cellular interactions as well as secretion of soluble factors. These stromal mediated mechanisms have been shown to impart drug-resistance to select chemotherapeutic agents [39, 40].

**Figure 4:**
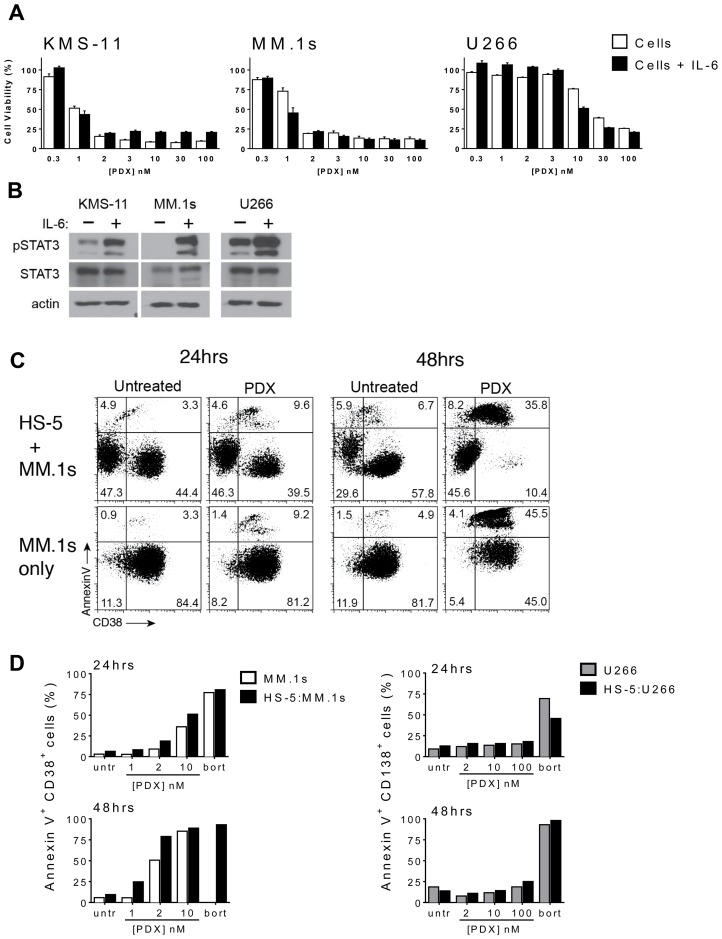
HMCLs retain sensitivity to PDX in the presence of microenvironment prosurvival factors. (**A**) A subset of HMCLs were cultured in the presence of IL-6 (5 ng/ml) for 24 hrs prior to incubation with increasing concentrations of PDX (0.3 nM–100 nM). Cell viability was assayed after 48 hrs of PDX exposure. The curves are normalized to untreated cells (100%) and bortezomib (10–50 nM) treated cells (0%). Data represent the mean ± SD of at least three experiments. (**B**) KMS-11, MM.1s and U266 cells were cultured for 24 hrs with or without IL-6 (5 ng/ml) and whole cells lysates were analyzed by western blot for phospho-STAT3 Tyr^705^ (pSTAT3), total STAT3 (STAT3). β-actin served as a loading control. (**C**) MM.1s and U266 cells were incubated on a monolayer of HS-5 bone marrow stroma-derived cells (BMSC) and incubated with increasing concentrations of PDX (MM.1s: 1 nM, 2 nM, 100 nM; U266: 2 nM, 10 nM, 100 nM). Dual color flow cytometry plots depicting apoptotic (annexin V^+^) MM.1s (CD38^+^) cells at 24 hrs and 48 hrs. (**D**) The full data set of the apoptotic HMCL population depicting MM.1s cells (AnnexinV^+^ CD38^+^) alone (*white*), U266 cells (AnnexinV^+^ CD138^+^) alone (*gray*) and HS-5: HMCL co-culture respectively (*black*). Untr = untreated vehicle control cells.

We examined the effects of IL-6 exposure, a principle cytokine important for myeloma cell proliferation and survival, on tumor cells treated with MTX and PDX. Co-incubation of the drug sensitive cell lines MM.1s and KMS-11 with IL-6 (5 ng/mL) and PDX did not cause a significant shift in the concentration dependent response, and did not change the IC_50_ value compared to cultures in the absence of cytokine (Figure 4A). Similarly, incubation with IL-6 did not sensitize the drug resistant cell line U266 to PDX (Figure 4A). IL-6 did invoke an on target response in each of the cell lines as demonstrated by the induction of STAT3 phosphorylation at Tyr^705^ in MM.1s, KMS-11 and U266 cells exposed to IL-6 (Figure 4B). It should be noted that while each myeloma cell line exhibited distinctly different basal levels of STAT3 activation [41, 42]; this did not correlate with sensitivity to PDX nor MTX.

As myeloma is highly dependent on cell - cell interactions and paracrine signaling provided by the bone marrow microenvironment, co-culture experiments were performed to determine the import of these variables on drug sensitivity. Co-culture with the transformed HS-5 BMSC line is thought to recapitulate aspects of the microenvironment influence and may mediate contact-mediated drug resistance in some settings. PDX-sensitive (MM.1s) and resistant (U266) myeloma cell line were plated onto a layer of HS-5 cells and co-cultured for up to 48 hours in the presence of increasing doses of PDX. Those myeloma cell lines plated without stroma served as a control. Myeloma cells were selected by staining for the expression of the lymphocytic surface markers, including CD38 (MM.1s) and CD138 (U266). Tumor cell viability was determined by the identification of the double positive CD138+/annexinV+ or CD38+/annexinV+ populations from the myeloma cell marker total subset (Figure 4D). The kinetics and concentration response to PDX were consistent with all the previous assays, confirming that these conditions did not change the patterns of sensitivity or resistance among the cell lines, nor did it affect the differential potency of the two drugs. These data suggest the protective effects of IL-6 and the bone marrow microenvironment seen with some agents does not alter patterns of PDX sensitivity.

### Sensitivity to pralatrexate correlates with RFC expression

**Figure 5:**
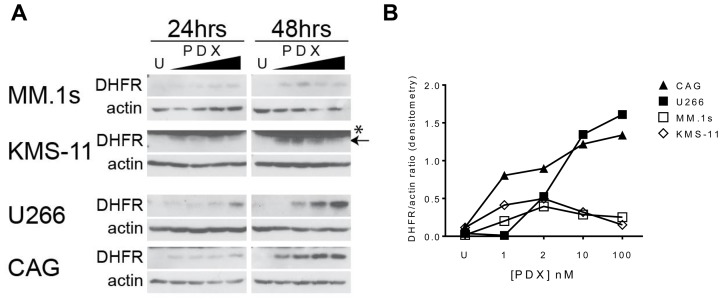
Antifolate-resistance in HMCLs correlates to the magnitude of DHFR protein upregulation in response to PDX. (**A**) PDX-sensitive cells MM.1s, KMS-11 and PDX-resistant cells U266, CAG were incubated with increasing concentrations of PDX (1, 2, 10, 100 nM) for 24 and 48 hrs. Whole cell lysates were run on a SDS-PAGE gel and protein expression analyzed by western blot. (**B**) The semi-quantitative densitometry data for the relative expression levels of DHFR (ratio of DHFR band intensity/beta-actin band intensity) for 48 hr samples in panel A. U = untreated cells, ^*^ denotes a non-specific band at 25 kDa in the KMS-11 & ARH-77 cell lines, proper band is below (*arrow*).

To identify discrete biomarkers of sensitivity and resistance to pralatrexate, we surveyed a panel of pharmacologic determinants established as possibly contributing to the phenotype. A subset of PDX-sensitive (MM.1s, KMS-11) and resistant (U266, CAG) myeloma cells were treated with increasing concentrations of PDX and expression of DHFR protein were quantitated by western blot over a 48-hour period (Figure 5A, 5B). Basal levels of DHFR protein in each cell line were low, and in some cases even undetectable, in comparison to the drug treated samples. This may reflect a generally lower rate of proliferation compared to other lymphoproliferative malignancies. As observed in Figure 5A, the concentration dependent stabilization of DHFR protein was found in both sensitive and resistant cell lines, increases in DHFR protein levels in response to drug were substantially higher in the PDX resistant cell lines (U266, CAG) compared to PDX-sensitive lines (MM.1s, KMS-11). This fits with the established features of MTX resistance, wherein amplification of DHFR, for example, strongly correlates with MTX resistance.

**Figure 6:**
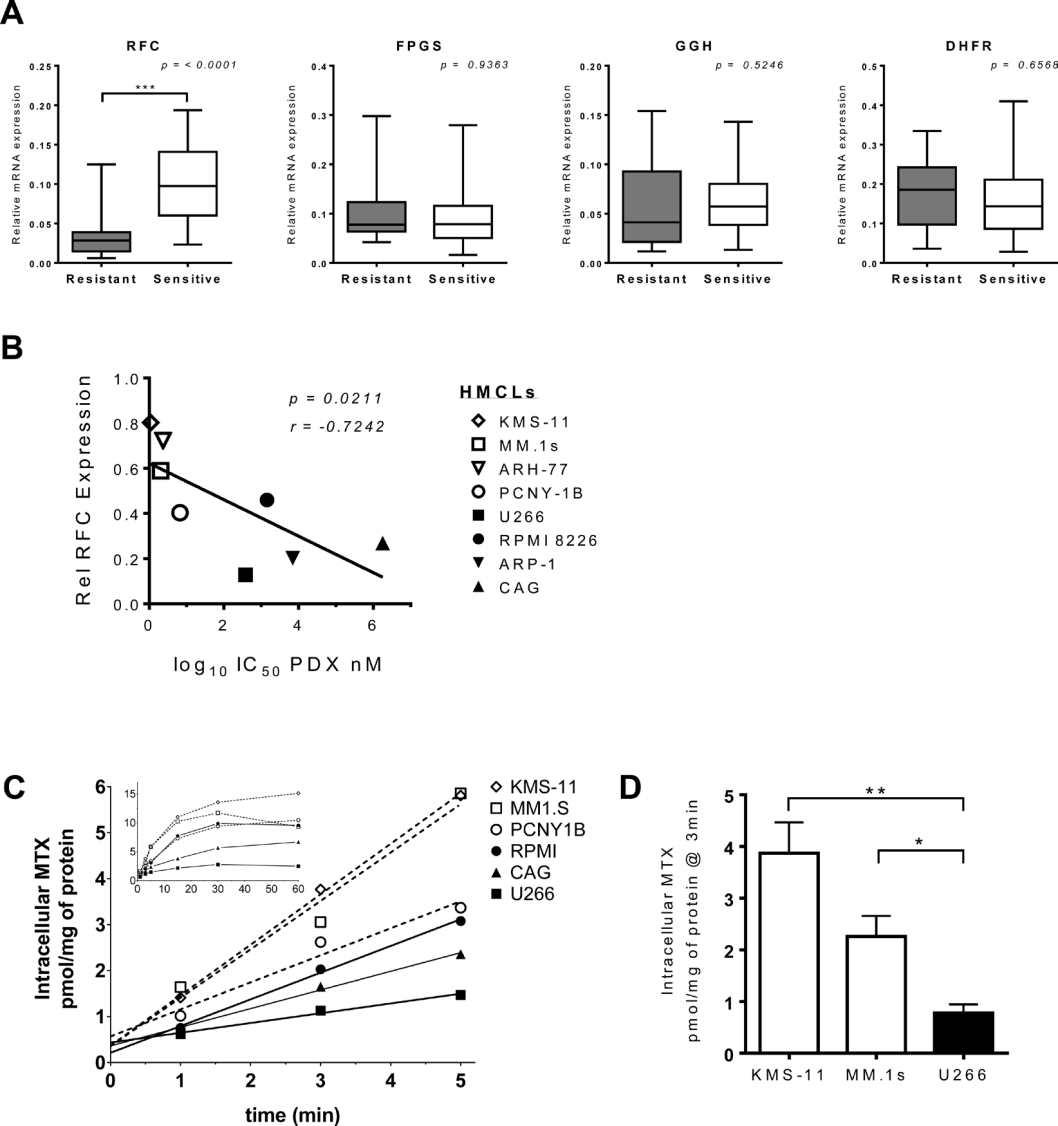
RFC expression and function correlate with PDX-sensitivity in HMCLs. (**A**) Relative mRNA expression of folate pathway genes in PDX-senstive (*white*) and PDX-resistant (*black*) HMCLs. The gene transcripts analyzed by RT-qPCR analysis were *RFC*, *FPGS*, *GGH* and *DHFR*. Eight cell lines made up the panel of HMCLs, four resistant: CAG, U266, ARP-1, RPMI 8226 and four sensitive: PCNY-1B, ARH-77, KMS-11 and MM.1s. Messenger RNA levels from each respective gene transcript were normalized to beta-actin and cyclophilin B. The data represent a minimum of three individual experiments. The box whisker plot demarcations: mean (*line*), box (*25th-75th percentile*), whiskers (*minimium and maximium*). The two-tailed *p* values were obtained through an unpaired Student *t*-test. (**B**) Correlation between *RFC* mRNA expression levels in 8 HMCL lines (*open symbols* = PDX-sensitive, *filled symbols* = PDX-resistant) and their respective IC_50_ values for PDX. The *p* value is one-tailed, *r* value = Pearson correlation coefficient. (**C**) The net uptake kinetics of MTX in a panel of resistant (*black, solid line*) and sensitive (*open, dashed line*) HMCLs. Cells were exposed to 1 μM MTX spiked with [^3^H-MTX] and samples were taken at 1, 2, 3, 5, 15, 30 and 60 min after the initial exposure. The main chart displays linear data obtained from 0-5 mins; the inset shows all data points 0–60 mins. Data are from a single experiment, which is representative of repeat studies. (**D**) Data compiled from a set of experiments comparing intracellular MTX level differences at 3 minutes after ^3^H-MTX incubation in KMS-11, MM.1s and U266 cells. The *p*-value was calculated by multiple *t*-test analysis and significance determined using the Holm-Sidak method (alpha = 5.0%) ^*^ *p* < 0.05, ^**^ *p* < 0.005.

To explore the significance of other pharmacologic and genetic determinants of resistance, we quantitated the expression levels of gene transcripts associated with antifolate sensitivity. The relative mRNA expression levels of four folate pathway genes (*RFC*, *GGH*, *FPGS* and *DHFR*) were examined in eight myeloma cell lines, including four resistant lines (ARP-1, CAG, RPMI 8228, U266) and four sensitive lines (ARH-77, KMS-11, MM.1s, PCNY-1B). While expression of each gene differed between the different cell lines (Supplementary Figure 3A), when the lines were grouped based on their pattern of sensitivity to PDX-sensitivity, a trend emerged (Figure 6A). As shown in Figure 6B, pralatrexate-resistant cell lines consistently expressed substantially lower levels of RFC mRNA compared to sensitive cell lines, a finding that was highly statistically significant (*p* < 0.0001). These data suggest RFC expression in myeloma tumor cells is the key biomarker of sensitivity to pralatrexate.

Efforts to corroborate the levels of RFC at the level of the protein were complicated by the fact that there are no reliable antibodies against RFC. Hence, we utilized a functional assay to quantitate radiolabeled MTX influx, as reported by Zhao *et al*. with an intent to correlate the rates of internalization with the pattern of pralatrexate and MTX sensitivity and RFC mRNA expression [43]. Cell lines were incubated with non-saturating extracellular concentrations of methotrexate spiked with [^3^H] MTX over one hour to measure the uptake of drug as a function of time. The transport was performed at pH 7.4 to minimize the contribution of endogenous proton-coupled folate transporter (PCFT) on MTX uptake [44, 45]. The equilibrium is achieved relatively quickly as demonstrated by the flattening of the slope of intracellular MTX influx over time (Figure 6C). Drug sensitive MM.1s and KMS-11 cells exhibited significant internalization of MTX as determined by intracellular concentrations of MTX. Conversely, the drug resistant U266 cells, which expressed relatively low RFC mRNA, demonstrated a limited capacity to internalize MTX. The differential in MTX intracellular concentration between the sensitive cells and U266 was highly statistically significant (Figure 6C, 6D; MM.1s v. U266 – *p* = 0.0297; KMS-11 vs U266 – *p* = 0.0078). Individual statistical analyses for each time point within the linear range demonstrated that the influx rate of MTX in pralatrexate-resistant and sensitive cell lines differed substantially (Supplementary Figure 3B, 3C). As expected, the drug resistant cell lines (U266, CAG and RPMI) exhibited lower intracellular concentrations of MTX compared to the sensitive cell lines (KMS-11, MM.1s and PCNY-1B; Figure 6C). Importantly, the results at the extremes of the data range demonstrate a direct correlation between increased RFC function and increased sensitivity to PDX in myeloma tumor cells.

## DISCUSSION

While multiple myeloma is not thought of as a disease sensitive to antifolates, it is clear there are a number of determinants that might influence the conclusion. First, methotrexate has been consistently shown to be inferior to pralatrexate, by at least a log-fold across all the biochemical and physiological assays studied to date. Hence, it is possible this class of drugs is ‘overlooked’ based upon suboptimal experiences with earlier analogs. Second, it is clear that at least RFC, and likely other pharmacologic determinant like DHFR and FPGS, can influence disease sensitivity to the class. It is likely that identifying a multiple myeloma ‘sensitive’ population based on RFC expression would enrich for patients likely to respond.

These data have consistently demonstrated that across all assays, there is a clear dichotomy among the myeloma cells studied: they are either highly sensitive to MTX and pralatrexate, or they are highly resistant to these agents. The classification of these two types of cells was corroborated across a variety of assays ranging from Cell Titer Glo to Annexin V and caspase 3 and 9 cleavage, to the cell cycle analysis. No cell line was found to be resistant in one assay and sensitive in another. There was a clear separation between the two phenotypes of myeloma cells.

Multiple myeloma is highly dependent on cytokine signaling pathways, be it IL-6, other paracrine pathways, or direct cell: cell mediated contact with the stromal microenvironment. It has been well established in a variety of myeloma models that all of these factors can contribute to drug resistance, and identifying drugs that maintain their activity irrespective of these stromal factors is an important goal in myeloma research. It has been demonstrated that the stromal environment plays an important role in cellular resistance to drugs including dexamethasone and doxorubicin. These factors did not have any impact on pralatrexate or methotrexate sensitivity in our analysis. Importantly, these data suggest that the potent cytotoxicity of pralatrexate is mediated independent of IL-6. Interestingly, IL-6 induced pSTAT3 expression, which has also been correlated with anti-apoptotic, pro-proliferation signaling pathways [46–48].

Differential expression of RFC appears to be a critical pharmacologic determinant in pralatrexate sensitivity in MM, which is notable since the drug was optimized for affinity to this transporter. Consistent with all the assay data, sensitive lines successfully internalized higher quantities of anti-folate (MTX), while resistant cell lines were found to have significantly lower intracellular concentrations of MTX. These internalization experiments correlated with the sensitivity pattern, RFC mRNA data and all the cytotoxicity data. Ideally, it would be valuable to know if RFC expression itself is prognostic, and whether a rapid assay could be developed to identify MM patient tumor cells as high versus low RFC expressers in a clinical setting. Gene expression profiling of tumor cells from patients may provide a means to study this hypothesis in a prospective manner.

In conclusion, we demonstrate that pralatrexate has promising pre-clinical activity in a subset of MM cell lines. Baseline RFC mRNA expression and induced expression of DHFR after exposure are functional pharmacologic biomarkers in this setting. Clinically, these data create a clinical trial scenario where pralatrexate could be studied in an all-commerce phase 2 study, with analysis of patient derived tissue for RFC and other pharmacologic determinants. This study could then be followed by a study where the biomarker of interest, in this case RFC, is used to screen patients for eligibility. This approach could allow for the identification of a novel drug in the disease, and establish a means to treat only those patients likely to benefit.

## MATERIALS AND METHODS

### Cell lines

The human myeloma cell lines used in these studies included U266, RPMI-8226 (obtained from American Tissue Culture Collection - ATCC), MM.1s, ARP-1, ARH-77, CAG, SK-MM2, KMS-11 (provided by S. Chen-Kiang, Weill Cornell Medical College, New York, NY) and PCNY-1B (provided by HJ Cho, Icahn School of Medicine at Mt. Sinai). All myeloma cell lines, save PCNY-1B, were cultured in RPMI-1640 (Life Technologies) supplemented with 10% fetal bovine serum (FBS; Sigma-Aldrich), and 20 μg/ml Gentamycin (Life Technologies). PCNY-1B was cultured in X-Vivo 15 (Lonza Walkersville, Inc.), supplemented with 10% pooled human serum (Omega Scientific, Inc.). The human bone-marrow stromal cell line HS-5 (provided by S. Chen-Kiang) was cultured in DMEM (ATCC) supplemented with 10% FBS and 20μg/ml Gentamycin. All cell cultures were maintained at 37° C with 5% CO2 in 95% relative humidity.

### Drugs and reagents

Pralatrexate was purchased from Selleckchem. All other drugs and chemical entities were purchased from Sigma Aldrich. Antibodies for Western blotting were obtained as follows: beta-actin (abcam ACTN05), caspase 3 (Santa Cruz #7272); (Cell Signaling #9662), caspase 9 (Signaling #9502), DHFR (Sigma WH0001714M1), Mcl-1 (Signaling #4572), Stat3 (Signaling #9132), Phospho-Stat3 (Signaling #9145), PARP (BD Biosciences #556362).

### Cell viability assay

Cell viability was measured using a luciferase-coupled ATP quantitation assay (CellTiter-Glo, Promega). Cells were plated at a concentration of 1.5 × 10^5^ cells/ml at a final volume of 200 μl per well in a 96-well plate. The assay plates were incubated for 24, 48 or 72 h at 37° C in the presence or absence of drug. In order to determine the impact of caspase cleavage on apoptosis cells were incubated with 50 mM pan caspase inhibitor, QVD-OPH (catalogue number OPH109, MP Biochemicals, Aurora, OH). At the appropriate time points, cells were harvested and transferred to opaque, white 96-well plates at which time the CellTiter-Glo reagent was added at a volumetric ratio of 1:1. The intensity of luminescence in the plates was measured using a SynergyH1 plate reader (BioTek).

### Apoptosis assay

Apoptotic cells were determined by Annexin-V+ using a staining method described previously [49]. Cells were resuspended in a small volume (100 μL) of 1× binding buffer (BD Bioscience), and incubated with Annexin V-FITC (BD Biosciences) and 5 μL of 7-Aminoactinomycin D (7-AAD; BD Biosciences) at room temperature, in the dark for 20 minutes. Untreated cells stained with Annexin V only or 7-AAD only served as single color controls. Cells were then fixed in 100μL of 4% paraformaldehyde (Sigma- Aldrich), collected by flow cytometry on a FACSCalibur (BD Bioscience) using CellQuest and analyzed by Flowjo.

### Western blot analysis

Protein lysates were prepared by freeze-thawing cells in lysis buffer (350 mmol/L NaCl, 20 mmol/L HEPES (pH 7.9), 0.2% NP-40, 1 mmol/L MgCl2, 1 mmol/L DTT, 20% glycerol, 2 mmol/L sodium orthovanadate, 10 mmol/L b-glycerol phosphate, and protease inhibitor (Calbiochem). Protein concentration was determined using the Bradford assay (Bio-Rad) according to the manufacturer’s instructions. Lysates (10 μg) was run on an SDS-PAGE gel and transferred to a polyvinylidene difluoride membrane (Millipore). Blocking and antibody dilutions were made in 5% nonfat dry milk (NFDM) in TBS-T [10 mmol/L Tris base, 150 mmol/L NaCl, 0.01% (v/ v) Tween 20; Sigma-Aldrich) or 5% bovine serum albumin (BSA). Blots were visualized with Supersignal West Femto Substrate (Thermo Scientific). Densitometry analysis was performed on ImageJ (NIH. Bethesda, MD) per the manufacturer’s recommendations using beta-actin as a reference control.

### Cell-cycle BrdU assay

The flow cytometry-based cell cycle progression assay was carried out as described by the manufacturer of BD BrdU FITC Assay (BD Biosciences). MM.1s cells (2.0 × 10^5^ cells/mL) were incubated with drug or vehicle for specified durations. One hour prior to collection of each time point the cells were incubated with BrdU (10 μM). After incubation the cells were washed (1× PBS), fixed, permeabilized and stained as described by the manufacturer. The DNA was co-stained with 7-AAD and the cells were visualized on a FACSCalibur cytometer.

### RNA isolation

Cell pellets were solubilized in 1 ml TRI reagent (MRC, Cincinnati, OH) and then sonicated for 10s on ice with a Sonic Dismembrator (Model 100 Fisher Scientific, Weltham MA). Each sample was mixed with 100 μl bromochlorophenol (MRC) and incubated at room temperature for 10 min, centrifuged at 14,000 × g, 4° C for 10 min. The aqueous phase was collected, and RNA was precipitated by adding 500μl isopropanol (Sigma-Aldrich, St. Louis, MO) and incubated at room temperature for 10 min, followed by centrifugation at 13,000 × g, 4° C for 15 min. The RNA pellet was washed with 75% ethanol (Sigma-Aldrich) and centrifuged again as in the previous step. The RNA pellet was air-dried and then re-suspended in 50–100 μl nuclease-free water and incubated at 70° C for 5 min. The concentration was determined by measuring the optical density of diluted samples at 260 nm in a Beckman Coulter DU530 spectrophotometer (Beckman Coulter Inc., Fullerton, CA).

### Gene expression analysis

Quantitative real-time RT-PCR (qRT-PCR) was carried out as previously described [50]. Taqman primer sets (Applied Biosciences) for the genes of interest in these experiments included the following: β-actin (Hs00181698_m1), cyclophilin B (Hx00168719_m1), SLC19a1 (Hs00953344_m1), FPGS (Hs00191956_m1), GGH (Hs00914163_m1) and DHFR (Hs00758822_s1). Relative expression was calculated against the geometric mean of the reference primers (β-actin and cyclophilin B) by the following formula: relative expression = 2-[ΔCt (sample) - ΔCt (reference)], where ΔCt = Ct (test) – Ct (baseline control).

### Radiolabeled MTX membrane flux assay

Cells were pelleted and washed twice in HBS buffer (20 mM HEPES, 140 mM NaCl, 5 mM KCl, 2 mM MgCl2, and 5 mM dextrose; adjusted with 1 N NaOH to achieve a pH of 7.4). Cells were resuspended in HBS buffer at the final density of 10–15 × 10e6 cells/ml, transferred in stirrer-glass tubes and incubated in a 37° C water bath for 20 min. Then, MTX spiked with [3H] MTX was added to a final concentration of 1 μM and uptake was performed at a pH 7.4. Aliquots from the cell suspension were collected over time and the reaction was stopped by injection of 10 volumes of ice-cold HBS buffer. The cells were washed and digested with 500 ml 0.2 N NaOH at 65° C for 45 min. Lysates were assessed for tritium on a liquid scintillation β-counter and protein concentration was determined by BCA assay (Thermo Fisher Scientific, Waltham, MA). Intracellular MTX was expressed as picomoles per milligram of protein.

### Statistical analyses

GraphPad Prism 5.0/6.0 was used to tabulate, chart and calculate all data and statistics. Unless noted all *p* values are obtained by implementing a paired Student’s *t*-test, all values yielding a *p* < 0.05 are considered significant values. Unless otherwise noted: ^*^ *p* < 0.05, ^**^ *p* < 0.01, ^***^ *p* < 0.001.

## SUPPLEMENTARY MATERIALS



## Abbreviations

PDX: pralatrexate
MTX: methotrexate
MM: multiple myeloma
RFC: reduced folate carrier
