# Supplementary material for: The anti-tumor activity of pralatrexate (PDX) correlates with the expression of RFC and DHFR mRNA in preclinical models of multiple myeloma
Source: Oncotarget. 2020 May 5;11(18):1576–89. doi: 10.18632/oncotarget.27516 (PMC7210016; doi:10.18632/oncotarget.27516)
Supplement: Supplementary file 1 [file oncotarget-11-1576-s001.pdf]

The anti-tumor activity of pralatrexate (PDX) correlates with the expression of RFC and DHFR mRNA in preclinical models of multiple myeloma

SUPPLEMENTARY MATERIALS

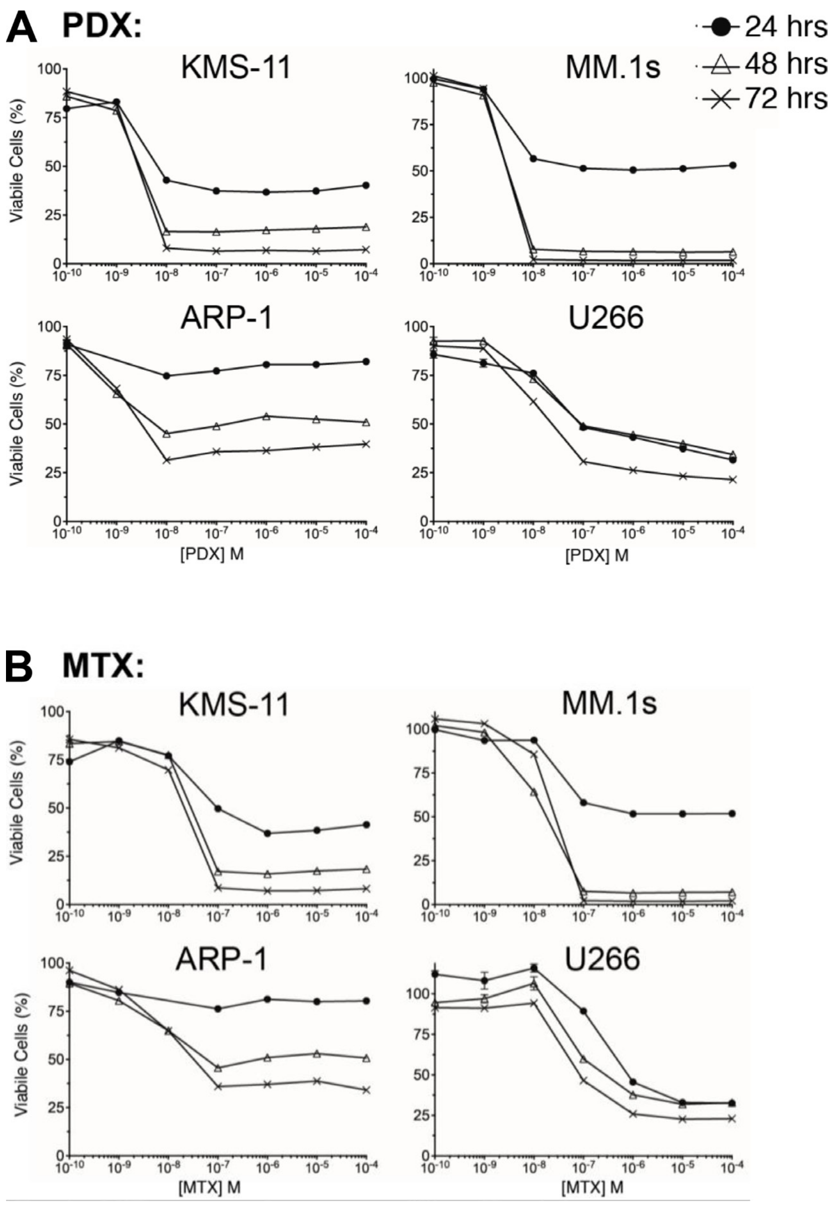

Supplementary Figure 1: Cell-viability curves demonstrate time and concentration dependence after treatment with antifolates in HMCLs. KMS-11, MM.1s, ARP-1 and U266 HMCLs were incubated with increasing concentrations of (**A**) pralatrexate (PDX) or (**B**) methotrexate (MTX) for 24, 48 and 72 hrs. Curves were generated using CellTiter Glo.

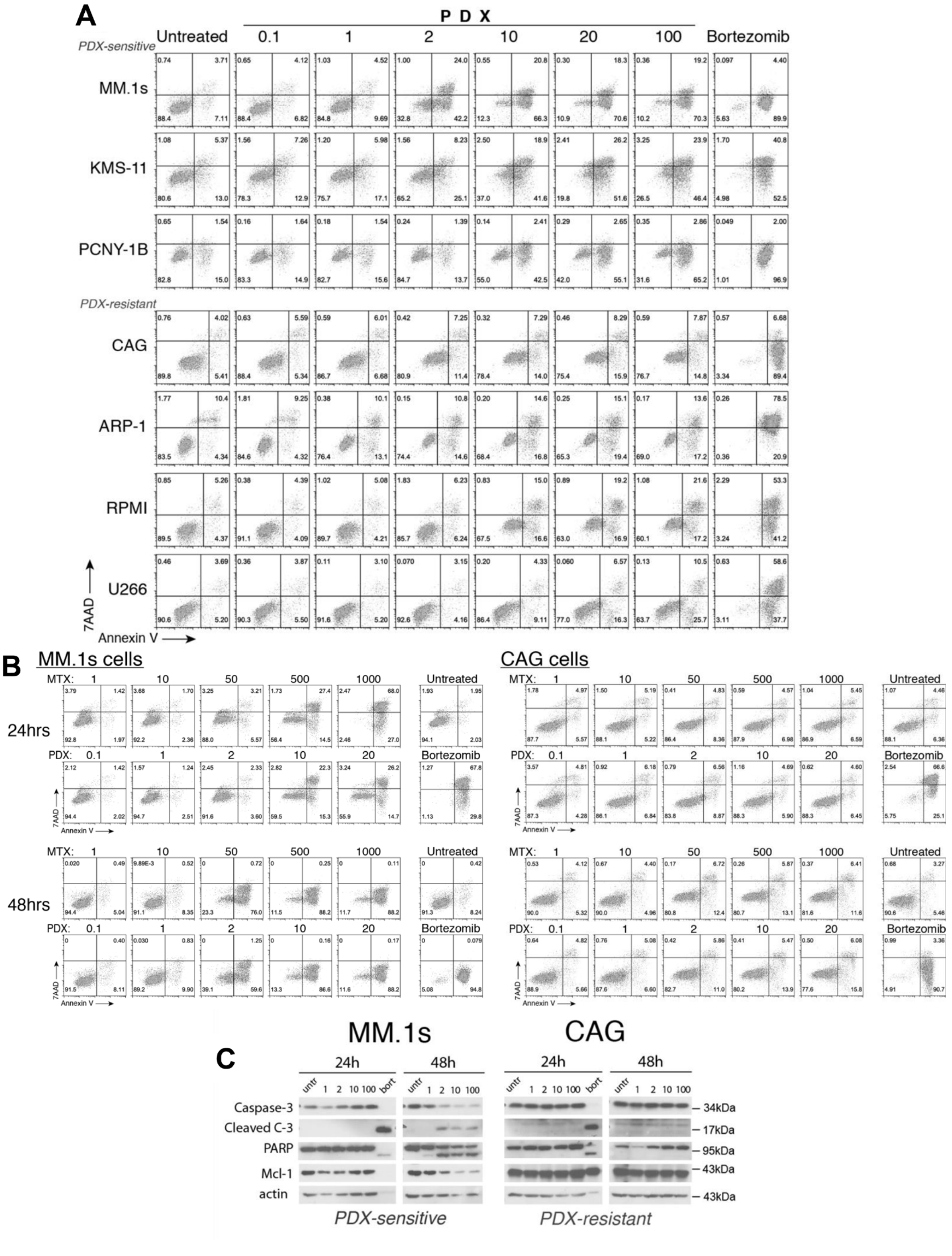

Supplementary Figure 2: Apoptosis induction observed in a panel of PDX-sensitive and PDX-resistant HMCLs. (**A**) A flow cytometry dot plot array shows 7AAD/αBrdU co-stained MM.1s, KMS-11, PCNY-1B, CAG, ARP-1, RPMI and U266 cells that were incubated with increasing concentrations of PDX (0.1–100 nM). Time point was taken at 48 hours post treatment. Untreated cells (*untr*) served as a negative control and bortezomib (10–50 nM) treated cells served as a positive control for apoptotic cell death. (**B**) A flow cytometry dot plot array shows 7AAD/ αBrdU co-stained MM.1s and CAG cells at 24 and 48 hours post treatment with MTX (1–1000 nM) or PDX (0.1–20 nM). Untreated cells (*untr*) served as a negative control and bortezomib (10–50 nM) treated cells served as a positive control for apoptotic cell death. (**C**) PDX-sensitive cells, MM.1s, and PDX-resistant cells, CAG, were incubated with increasing concentrations of PDX (1, 2, 10, 100 nM) for 24 and 48 hrs. Whole cell lysates were run on a SDS-PAGE gel and protein expression analyzed by western blot.

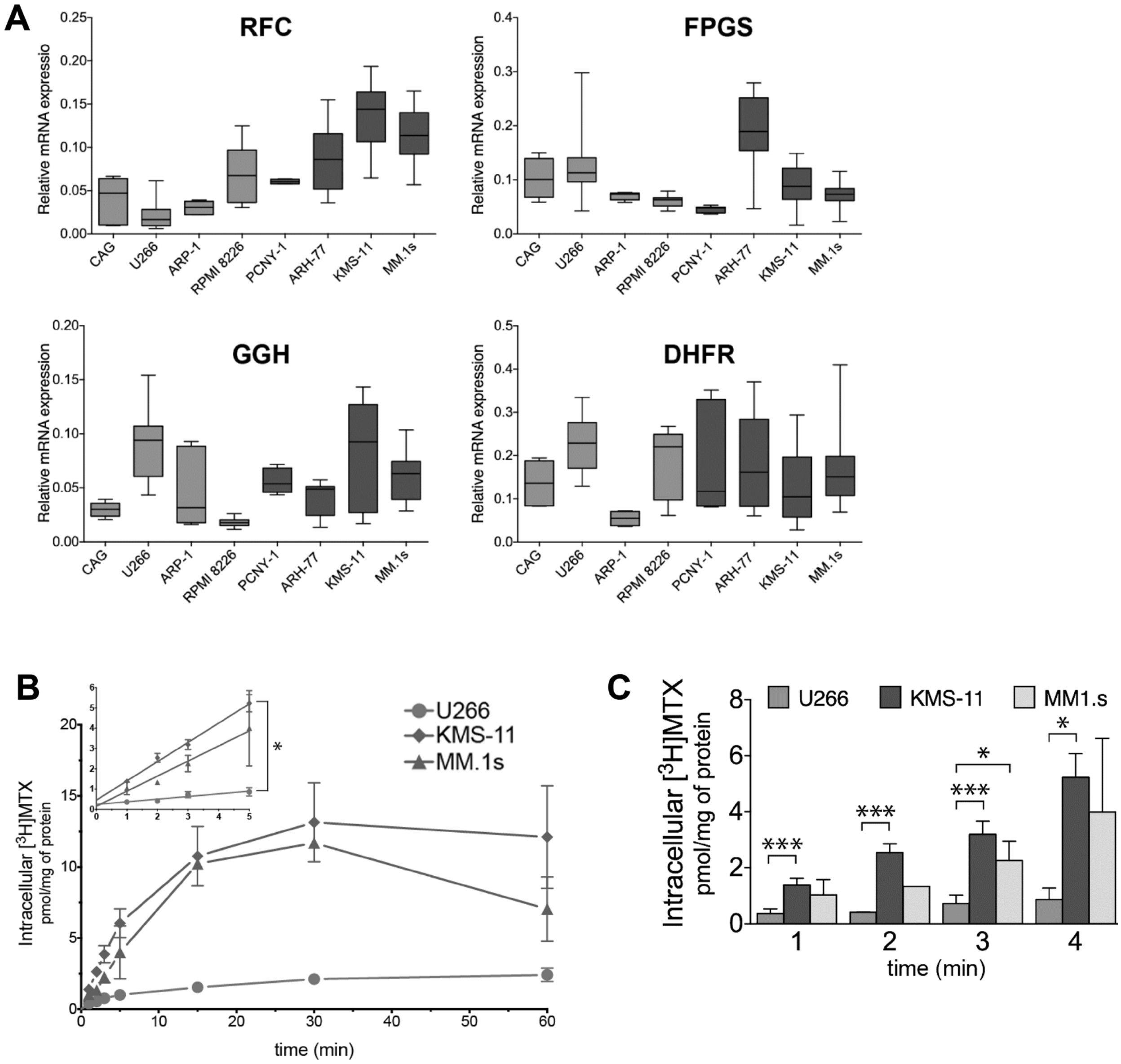

Supplementary Figure 3: Analysis of folate pathway genes reveals differential expression patterns linked to PDX-sensitivity in HMCLs. (**A**) Relative mRNA expression of folate pathway genes in PDX-senstive (black) and PDX-resistant (*gray*) HMCLs. The gene transcripts analyzed by RT-qPCR analysis were *RFC, FPGS, GGH* and *DHFR*. (**B**) The net uptake kinetics of MTX in a panel of resistant and sensitive HMCLs (U266, KMS-11 and MM.1s). Cells were exposed to 1 μM MTX spiked with [^{3}H-MTX] and samples were taken at 1, 2, 3, 5, 15, 30 and 60 min after the initial exposure. (**C**) Data compiled from a set of experiments comparing intracellular MTX level differences at 1, 2, 3 and 4 minutes after ^{3}H-MTX incubation in KMS-11, MM.1s and U266 cells. The *p*-value was calculated by multiple *t*-test analysis and significance determined using the Holm-Sidak method (alpha = 5.0%) $^{*}p < 0.05$, $^{**}p < 0.005$.
